# Contamination level, sources, and health risk of polycyclic aromatic hydrocarbons in suburban vegetable field soils of Changchun, Northeast China

**DOI:** 10.1038/s41598-022-15285-5

**Published:** 2022-07-04

**Authors:** Zhengwu Cui, Yang Wang, Liansheng Du, Yong Yu

**Affiliations:** 1 Northeast Institute of Geography and Agroecology, Chinese Academy of Sciences, Changchun, 130102 China; 2 University of Chinese Academy of Sciences, Beijing, 100049 China; 3 Shanghai Huipeng Environmental Technology Co., Ltd., Shanghai, 200333 China

**Keywords:** Environmental chemistry, Environmental monitoring, Geochemistry, Environmental sciences

## Abstract

Polycyclic aromatic hydrocarbons (PAHs) are a class of persistent organic pollutants. With the expansion of the city, the suburban environment is being increasingly polluted by PAHs, which pose a huge potential risk for suburban agriculture. Therefore, we conducted a survey focusing on the pollution level, sources, and risk of PAHs in Changchun suburban vegetable soils, Northeast China. The total concentrations of 16 PAHs (Σ_16_PAHs) in soils were between 2338.2 and 15,200 ng g^−1^ (mean 6778.1 ng g^−1^), which were significantly higher than those in most other cities. High molecular weight PAHs were the major components, which occupied over 85.63% of all PAHs. Seven potential carcinogenic PAHs accounted for 56.96% of the Σ_16_PAHs. Source apportionment results based on the ratio of PAH isomers and principal components analysis showed that PAHs were primarily derived from pyrolysis sources, such as biomass/coal combustion, traffic emissions, and petroleum. Ecological risk values of PAHs were between effects range-low (ERL) and effects range-median (ERM), which might cause occasionally ecological risks in the suburbs. According to the incremental lifetime cancer risk assessment results, the health risks to the exposed population were in the acceptable level, with dermal contact and ingestion being the predominant exposure pathway.

## Introduction

Rapid industrialization and urbanization have increased environmental pollution in recent decades, especially that caused by persistent organic pollutants (POPs) with high toxicity, persistence, bioaccumulation, and long-distance migration. Polycyclic aromatic hydrocarbons (PAHs) are typical POPs derived from natural and anthropogenic sources. The anthropogenic sources of PAHs are the primary sources and very extensive, such as mobile sources, civil combustion sources and industrial sources, which account for 50.5%, 41.6%, and 7.9% of the total emissions of PAHs, respectively^1^. PAHs are easily adsorbed in suspended particles and aerosols, and can enter into the soil through the dry and wet deposition^2^. Due to hydrophobicity and stability, PAHs can be adsorbed by soil organic matter, causing toxicity to soil microorganisms and plants, and further affecting the soil ecological function^3^. The soil-to-plant transfer is the main pathway via which people are exposed to the soil PAHs^4^. PAHs in soil can be absorbed by the roots and transferred to plant tissues, and then accumulated in the human body through the food chain. Studies have revealed that PAHs can cause pathological changes in human organs, such as the respiratory tract, kidney, liver, and even the endocrine system, reproductive system, and neural system^5–7^.

Vegetables contain various nutrients, such as vitamins, proteins, minerals, and carbohydrates, which play an important role in the human diet. As vegetables can be produced in small- or pilot-scale fields with a short growth cycle, it is very common to plant vegetables in suburban areas^8, 9^. The surge in urban population has promoted the rapid development of suburbs, and the changes in industrial activities and living environment in the suburbs have had a tremendous impact on agricultural production^10^. Urban expansion has resulted in the occupation of a large amount of arable land in the suburbs of the city for the relocation of factories or the construction of highway and new residential quarters, which make the remaining vegetable fields closer to pollution sources such as factories, roads, and residential areas, and even surrounded by buildings^11^. PAHs can enter the suburban agricultural soils from different sources, resulting in the accumulation of pollutants, affecting soil quality and human health^5^.

As the central city of the Northeast Asian economic circle, Changchun plays an important role in economic growth, urbanization, and international trade. In recent years, with the continuous development of urbanization, the land use patterns in the suburbs of Changchun have undergone significant changes. According to the Changchun statistical yearbook 2010 to 2020 (http://tjj.changchun.gov.cn/ztlm/tjnj/), the urban area in 2019 was 7293 km^2^ which was approximately 1.5-fold greater than that in 2009. In order to meet the living needs of the rapidly growing urban population, a large number of residential quarters and supporting thermal power plants or heating facilities have been built in the suburban areas, making the suburban living areas and the original industrial enterprise areas staggered or interlapped distribution. Meanwhile, private cars have become the main means of transportation due to being far away from the city center. These mobile sources, civil combustion sources and industrial sources have formed a comprehensive pollution effect in suburban areas, resulting in a large accumulation of PAHs in the soil, especially a high ecological risk for suburban vegetable soil. Earlier studies on pollutants in suburban vegetable soils of Changchun have mostly focused on heavy metals (HMs) and organochlorine pesticides (OCPs)^12–14^, while studies on PAHs are scarce. The comprehensive pollution level of PAHs in agricultural soils along the main roads in Changchun were evaluated, and found that over 90% of the sample points were above the light pollution level^15^. Moreover, nearly 30% of the samples were at the “high severity” or more serious ecological risk level^16^. Similar results were also obtained in snowpack and indoor dust^17, 18^. The above results showed that the potential ecological risks of PAHs were widespread in the suburbs of Changchun, but the overall quality of suburban vegetable fields was still not specified. Therefore, identifying the pollution level, sources and risks of PAHs in suburban vegetable soils is necessary, helping to establish effective risk management. Toward this, the levels of PAHs in vegetable soils of the Changchun suburb and their potential sources were investigated and the toxicological risks were determined.

## Materials and methods

### Study site and sampling

**Figure 1:**
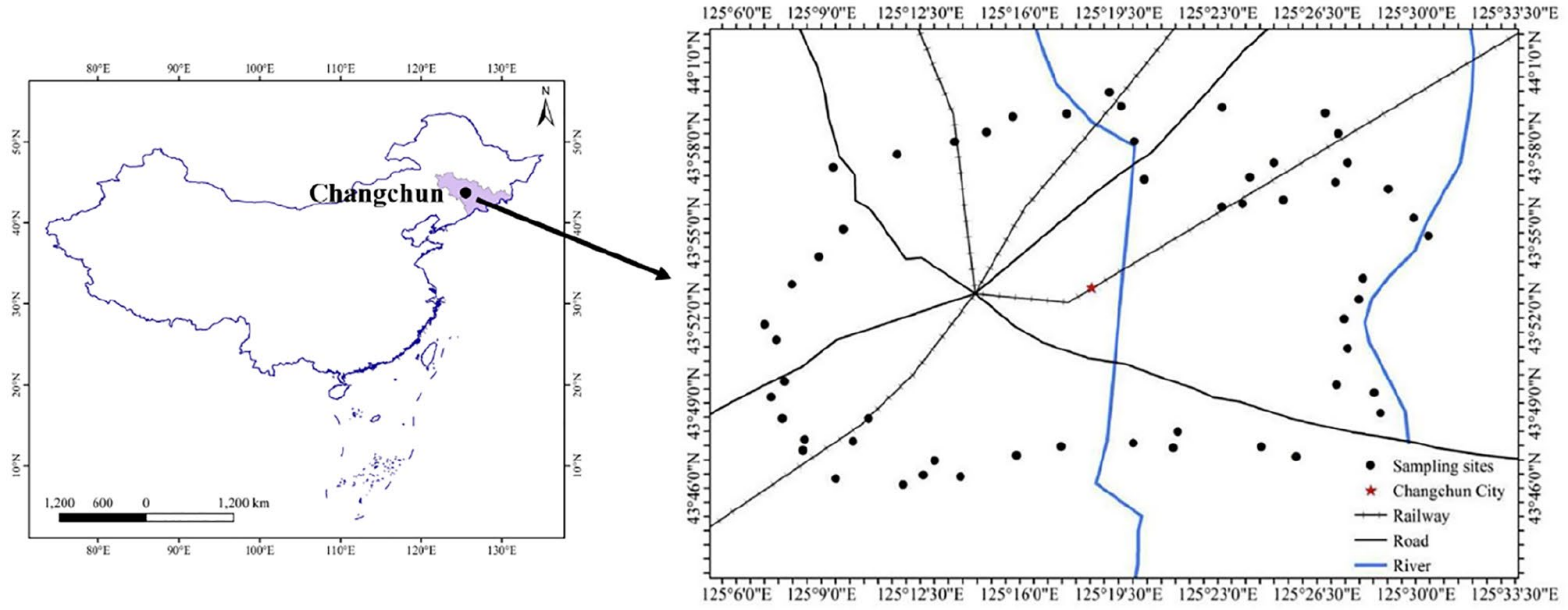
Study area and soil sampling sites.

Changchun, between north latitude 43°05′–45°15′ and east longitude 124°18′–127°05′, is the capital of Jilin Province and an important commodity grain base and industrial base in China. Changchun belongs to the continental monsoon climate with an annual average temperature of 4.8 °C. The main soil type is typical phaeozem suitable for reclamation and cultivation. Vegetable cultivated area in Changchun suburbs is 3617 hm^2^, with an annual yield of 9.8 × 10^4^ tons. 51 soil samples were collected in the spring of 2018. The probable geographic coordinates of the pre-set sampling points were imported into the GPS navigator, following which the sampling points were marked in the actual sampling site (Fig. 1). Four or five surface soil (0–10 cm) were collected at each point as the sub-samples by using the plum blossom method and then mixed to get representative samples. Approximately 500 g of soil was taken from each site and then stored in brown glass bottles after removing the stone, grass, or leaves, and other debris.

### Sample extraction and analysis

The sixteen USEPA priority PAHs analyzed in soil samples were: naphthalene (Nap), acenaphthylene (Acy), acenaphthene (Ace), fluorene (Flu), phenanthrene (Phe), anthracene (Ant), fluoranthene (Fla), pyrene (Pyr), benz[a]anthracene (BaA), chrysene (Chr), benzo[b]fluoranthene (BbF), benzo[k]fluoranthene (BkF), benzo[a]pyrene (BaP), Indeno[1,2,3-cd] pyrene (Inp), dibenz[a,h]anthracene (Daha), and benzo[g,h,i]perylene (Bghip)^19^. Ultrasonic extraction of PAHs and cleanup procedures followed the previous method^15, 20^. All PAH concentrations were reported on a dry weight basis.

*Extraction* 2.0 g of soil samples (moisture content measured) were mixed with 5 g anhydrous sodium sulfate and 15 mL solvent (n-hexane: acetone = 1:1, v/v) in a centrifuge tube and were ultrasonically extracted for 15 min. The extracts were collected in a round bottom flask after centrifugation for 10 min at 4500 rpm. Extraction processes were carried out three times, and a total of 45 mL of extracts were obtained. The extracts were rotary evaporated to near dry at 40 °C, and then re-dissolved in 2 mL n-hexane.

*Purification* The concentrated extracts were purified by Florisil column and eluted with 5 mL acetone/n-hexane mixtures (V:V = 1:9). The eluent was concentrated to near dry and diluted to 2 mL with dichloromethane.

*Determination* The 16 PAHs were examined using GC equipped with a flame ionization detector and RTX@-5 capillary column. The oven temperature program of GC was started at 50 °C (1 min), ramped to 200 °C at a rate of 25 °C/min (1 min), then further ramped to 280 °C at 10 °C/min (held for 20 min).

### Quality control

All analytical data were subject to strict quality control. Procedural blanks, spiked blanks, and sample duplicates were conducted for quality assurance and control. Spiked blank results showed that the recoveries of 16 PAHs in any sample were within the acceptable range of 75–110%, which met the detection requirements for PAHs. The five-point external calibration method was applied in the sample analysis, and a linear relationship with R^2^ > 0.99 was obtained. The method detection limits (MDLs) of 16 PAHs ranged from 0.5 to 1.0 ng g^−1^.

### Potential health risk

Health risk assessment associates the probability of toxic effects with the contaminant degrees in the environment. Prior to comparison with soil quality standards, the PAHs concentrations in the soil are usually converted to Bap equivalents. This method has been widely used in many studies to evaluate the PAHs cancer risk^15, 21^.

The toxic equivalent concentration (TEQ_BaP_) of PAHs was calculated by using the following equation:

1 $$TEQ_{BaP} = \sum\limits_{1}^{16} {TEF_{i} \times C_{PAHi} }$$

where *C*_*PAHi*_ is the concentration of PAH congener i in the soil and *TEF*_*i*_ is the toxicity equivalency factor (TEF) of PAH congener i.

The Incremental Lifetime Cancer Risk (ILCR) was applied to evaluate the comprehensive health risk of pollutants with ingestion, dermal contact, and inhalation of three different exposure pathways. Based on the USEPA standard model^22^, their probabilities were calculated using formulas (2)–(5):

2 $$ILCR_{ing} = \frac{{CS \times (CSF_{ing} \times \sqrt[3]{BW/70}) \times IR_{ing} \times EF \times ED}}{{BW \times AT \times 10^{6} }}$$

3 $$ILCR_{der} = \frac{{CS \times (CSF_{der} \times \sqrt[3]{BW/70}) \times SA \times AF \times ABS \times EF \times ED}}{{BW \times AT \times 10^{6} }}$$

4 $$ILCR_{inh} = \frac{{CS \times (CSF_{inh} \times \sqrt[3]{BW/70}) \times IR_{inh} \times EF \times ED}}{BW \times AT \times PEF}$$

5 $$ILCRs = ILCR_{ing} + ILCR_{der} + ILCR_{inh}$$

where *CS* is the sum concentrations of carcinogenic PAHs converted by toxic equivalent. Adult farmers in this study area were considered to be the direct receptors of PAHs because they spent time on cultivating vegetables in Changchun suburbs. The detailed parameters are listed in Table S1.

### Consent to publish

Yes.

## Results and discussion

### Concentration of PAHs in suburban vegetable soils

Descriptive statistics of PAHs concentrations in suburban vegetable soils of Changchun were presented in Table 1. Total concentrations of 16 PAHs (Σ_16_PAHs) in soils varied from 2338.2 to 15,200 ng g^−1^ (mean 6778.1 ng g^−1^) with a detection rate of 100%. The concentration of PAHs was considerably higher than that in other cities elsewhere. The average concentration of Σ_16_PAHs was 5.5, 2.0, 3.4, 2.0, 2.5, and 2.1-fold of that in Beijing^23^, Nanjing^24^, Shanghai^25^, Lanzhou^26^, China, Lisbon, Portugal^27^, and Orlando, USA^28^, respectively. International Agency for Research on Cancer has categorized 7 isomers, which are BaA, Chr, Bbf, Bkf, Bap, Daha and Inp, as mutagenic and carcinogenic pollutants^29, 30^. Total concentrations of 7 carcinogenic PAHs (Σ_7c_PAHs) were between 742.23 and 9526.1 ng g^−1^ (mean 4615.7 ng g^−1^) which accounted for about 56.96% of Σ_16_PAHs.

**Table 1:** Descriptive statistics of PAHs in soils of the studied region (ng g^−1^, n = 51).

PAHs	Abbreviation	Aromatic ring	Concentration (ng g^−1^)						
			Min	Max	Mean	Median	SD	CV (%)	Composition (%)
Naphthalene	Nap	2	3.000	230.0	33.73	24.58	35.00	103.8	0.50
Acenaphthylene	Acy	3	7.000	619.1	86.65	63.89	95.60	110.3	1.28
Acenaphthene	Ace	3	10.29	239.3	46.67	28.07	47.09	100.9	0.69
Fluorene	Flu	3	8.440	962.0	88.84	55.02	144.5	162.7	1.31
Phenanthrene	Phe	3	56.70	1499.6	472.6	226.4	433.0	91.62	6.97
Anthracene	Ant	3	44.17	776.0	245.7	169.7	196.5	79.98	3.62
Fluoranthene	Fla	4	86.69	1593.7	372.0	274.9	285.5	76.75	5.49
Pyrene	Pyr	4	163.7	1388.6	593.2	532.0	297.2	50.10	8.75
*Benzo(a) anthracene	BaA	4	133.4	2016.2	480.4	388.9	394.2	82.07	7.09
*Chrysene	Chr	4	80.66	6179.9	1718.7	980.2	1780.4	103.6	25.36
*Benzo(b) fluoranthene	BbF	5	33.19	1223.5	338.7	252.3	276.2	81.55	5.00
*Benzo(k) fluoranthene	BkF	5	7.700	3184.8	459.0	231.6	593.0	129.2	6.77
*Benzo(a) pyrene	BaP	5	17.44	908.2	330.5	244.7	241.5	73.08	4.88
Dibenzo(a,h) anthracene	Daha	5	8.070	1210.8	222.9	124.6	265.1	118.9	3.29
*Indeno(1,2,3-cd) pyrene	InP	6	8.440	1577.6	310.5	105.5	382.3	123.1	4.58
*Benzo(g,h,i) perylene	BghiP	6	201.5	2304.1	978.1	960.3	383.6	39.22	14.43
	Σ_16_PAHs^a^		2338.2	15,200	6778.1	6493.5	3267.8		
	Σ_7c_PAHs^b^		742.23	9526.1	3860.6	3384.3	2361.3		56.96
	LMW		189.59	3239.6	974.2	735.7	659.1		14.37
	HMW		1620.9	13,700	5803.9	5622.5	2884.5		85.63

*SD* standard deviation, *CV* coefficient of variation.

^a^Σ_16_PAHs: total concentrations of 16 PAH.

^b^Σ_7c_PAHs: concentrations of 7 carcinogenic PAHs (BaA, Chr, BbF, BkF, BaP, InP, DahA).

PAHs containing four or more rings are defined as high molecular weight (HMW) PAHs, while others are considered to be low molecular weight (LMW) PAHs^31^. Based on the average concentrations, the contribution of HMW PAHs to Σ_16_PAHs was 85.63% (46.69% for 4-ring, 19.94% for 5-ring, and 19.01% for 6-ring PAHs), while 3-ring PAHs contributed about 13.87%, and the 2-ring PAHs accounted for about 0.50% (Table 1). These results were similar to those reported previously for the proportional distribution of PAHs. The concentration of HMW PAHs were more than 2 times that of LMW PAHs in soils collected from an industrial city in South Korea^32^. Four–six ring PAHs accounted for more than 60% of the total PAHs in the vicinity agricultural soils of a chemical plant in China^33^. In general, due to the high volatility, LMW PAHs mainly exist in the gas phase and can undergo photochemical degradation during atmospheric transport^34^. Nevertheless, most of the HMW PAHs can adhere to the particles and accumulated in the soil through the dry and wet deposition^35^. The abundance of HWM PAHs in soil indicated that the study area had been exposed to PAHs for a long time, and the PAHs may originate from fossil fuel combustion and traffic emissions^36^.

### Source apportionment

Numerous studies have proved that the sources of PAHs in soil can be identified by the ratio of isomers^20, 26^. In this study, the major sources of PAHs could be derived from five characteristic ratios^37^ (Table S2). Based on our results, PAHs of pyrogenic origin obtained from petroleum and biomass/coal combustion contribute mostly to the vegetable soils of Changchun suburb^34^.

Principal component analysis (PCA) has been successfully used for the source apportionment of PAHs^38^, which is an effective tool for quantitatively assessing the contribution of various sources of PAH contamination^39^. As presented in Table 2, 81.80% variance in the statistical data was explained by five components (PC1, PC2, PC3, PC4, and PC5).

**Table 2:** Factor analysis scores following Varimax rotation for all PAHs (factor loadings > 0.5 are shown in bold).

PAHs	Component				
	PC1	PC2	PC3	PC4	PC5
Nap	**0.891**	0.167	0.106	0.097	0.142
Acy	**0.864**	− 0.007	− 0.008	0.066	0.234
Ace	0.404	0.143	**0.691**	0.091	0.477
Flu	0.158	0.094	**0.891**	0.027	0.251
Phe	0.070	0.052	0.161	− 0.212	**0.827**
Ant	0.341	0.188	0.447	**0.650**	− 0.043
Fla	0.254	0.222	0.146	0.467	**0.687**
Pyr	**0.741**	0.090	0.447	− 0.012	− 0.141
BaA	**0.573**	0.351	0.345	0.080	**0.524**
Chr	− 0.032	− 0.268	− 0.179	**0.888**	− 0.040
Bbf	**0.653**	0.370	0.385	0.267	0.091
Bkf	− 0.029	**0.864**	0.003	− 0.207	0.167
Bap	**0.627**	0.494	0.423	0.100	0.255
Daha	0.132	0.489	0.313	**0.607**	0.030
Inp	0.244	**0.730**	0.454	0.120	− 0.071
Bghip	0.246	**0.782**	0.020	0.107	0.133
Eigenvalue	3.753	2.857	2.511	2.023	1.945
Total variance (%)	23.46	17.86	15.69	12.64	12.15
Cumulative variance (%)	23.46	41.31	57.01	69.65	81.80

PC1 explained 23.46% of the total variance, and Nap, Acy, Pyr, BaA, Bbf, and Bap were mainly loaded on this component. Pyr and BaP are indicators of coal combustion, while BbF is the component of fossil fuel combustion, and the combustion of natural gas is considered to be the source of BaA^40^. The 2–3 ring PAHs (Nap and Acy) might be derived from petroleum under low temperature or incomplete combustion^41^. The second component (PC2) explained 17.86% of the total variance with the primary contributor of Bkf, Inp, and Bghip. Bkf and BghiP are indicators of diesel-powered vehicles^40^. Inp and BghiP have been defined as the tracers of vehicle source^42^. Thus, PC2 was attributed to the traffic emission. The third and fourth components were highly loaded with Ace and Flu (PC3), and Ant, Chr, and Daha (PC4), respectively. These compounds are typically related to combustion. In particular, Ace is the dominant PAH in coal and wood combustion^43^. It is believed that Flu is related to coke combustion^41^. Ant and Chr are fingerprints of wood burning, while Chr and Daha are the specific products of biomass and coke oven combustion^44^. Consequently, these two components could be ascribed to the residential combustion of biomass and coal. The last factor was predominated by Phe, Fla, and BaA, which are the dominants in coal-fired industrial emissions such as power plants, industrial boilers and residential heating^45^.

Winter average temperature of Changchun ranges from − 19.7 to − 9.6 °C with a lowest temperature nearly to − 40 °C^12^. The heating period here is from the end of October to the beginning of April of the following year, which can last for about half a year. In most urban areas and some suburbs, heating mainly relies on coal-fired boilers for central heating. Moreover, in some suburban areas, residents still rely on burning coal or straw, firewood, and other biomass. The massive use of coal and biomass fuels would be one of the main contributors for PAHs accumulation in suburban soils throughout the heating period. The number of motor vehicles in Changchun has increased significantly with urbanization, and as of 2019, the number had exceeded 2.0 million. Therefore, vehicle emissions considerably impact PAH pollutants in the suburban area. In addition, as an industrial city in China, Changchun consumes a large amount of fuel for industrial production every year. Thus, to guarantee people's living conditions and the needs of industrial development, a large quantity of fossil fuel, biomass, and petroleum will be used. The PAH compounds generated in the above processes were released into the atmosphere, and then entered into the soil with the dry and wet deposition.

### Ecological risk

At present, there is no unified standard method for ecological risk assessment of soil PAHs. Sediment quality guidelines (SQGs) were proposed to evaluate the ecological risk of PAHs in sediments^46^, and this method has been commonly applied to evaluate the ecological risk of soil PAHs^47^. The ecological risks can be divided into three levels based on the effects range-low (ERL) and effects range-median (ERM): C_PAHs_ < ERL, negative ecological effects rarely occur; ERL ≤ C_PAHs_ ≤ ERM, negative ecological effects occasionally occur; C_PAHs_ > ERM, negative ecological effects often occur^46, 47^.

The comparisons of measured PAHs concentrations with ERL and ERM values were shown in Table 3. Concentrations of 12 individual PAH, LMW PAHs, HMW PAHs and total 12 PAHs (Σ_12_PAHs) exceeded the corresponding ERL and ERM values with different proportions. The average concentration of ∑_12_PAHs was between ERL and ERM, and the proportions of ∑_12_PAHs < ERL, ERL ≤ ∑_12_PAHs < ERM, and ∑_12_PAHs ≥ ERM were 47.06%, 52.94%, and 0.00%, respectively. Moreover, mean concentrations of both LMW PAHs and HMW PAHs were between ERL and ERM with 68.63% and 78.43% of samples exceeding the ERL values, respectively. For individual PAH, except for Nap, Pyr, Fla, and Bap were below the ERL, the average concentrations of remaining PAHs were between the ERL and ERM. Daha (29.41%) and Chr (19.61%) had the highest proportions exceeding the ERM value, indicating that these two pollutants had the greatest negative effects on the ecosystem. The above results showed that adverse ecological effects of PAHs might occasionally occur in the suburbs of Changchun, which should be paid enough attention.

**Table 3:** Comparison of PAHs contents with ERL and ERM guideline values (ng g^−1^).

Pollutant	Mean	ERL	ERM	Composition of C_PAH_ < ERL (%)	Composition of ERL ≤ C_PAH_ < ERM (%)	Composition of C_PAH_ ≥ ERM (%)
Nap	33.73	160	2100	98.04	1.96	0.00
Acy	86.65	44	640	27.45	72.55	0.00
Ace	46.67	16	500	17.65	82.35	0.00
Flu	88.84	19	540	1.96	96.08	1.96
Phe	472.6	240	1500	50.98	49.02	0.00
Ant	245.7	85.3	1100	29.41	70.59	0.00
Fla	372.0	600	5100	82.35	17.65	0.00
Pyr	593.2	665	2600	64.71	35.29	0.00
Chr	1718.7	384	2800	31.37	49.02	19.61
BaA	480.4	261	1600	31.37	64.71	3.92
Bap	330.5	430	1600	66.67	33.33	0.00
Daha	222.9	63.4	260	45.10	25.49	29.41
Σ_12_PAHs	4691.9	4022	44,792	47.06	52.94	0.00
LMW PAHs	974.2	552	3160	31.37	66.67	1.96
HMW PAHs	3717.7	1700	9600	21.57	78.43	0.00

### Potential health risk

#### Toxicity equivalent factor (TEF)

The TEF values for PAHs and the corresponding TEQ_BaP_ concentrations were presented in Table 4. The TEQ_BaP_ concentrations of Σ_16_PAHs and Σ_7c_PAHs in soil samples were 82.75–2320 ng g^−1^ (mean 743.4 ng g^−1^) and 63.40–1492 ng g^−1^ (mean 516.3 ng g^−1^), respectively. Σ_7c_PAHs was the main contributor to the total carcinogenic potency of PAHs, accounting for 69.5% of the total TEQ_BaP_. The proportions of different PAHs in total TEQ_BaP_ were different, which were arranged in the descending order: BaP (44.46%) > Daha (29.99%) > BaA (6.46%) > BkF (6.17%) > Bbf (4.56%) > InP (4.18%) > Chr (2.31%) > BghiP (1.32%). The average TEQ_BaP_ concentration of the Σ_16_PAHs in the suburban soil samples was under the World Health Organization (WHO) threshold (1000 ng g^−1^)^19^. Although the average value was at the safe level, 29.4% of the collected soil samples were still found to be higher than the standard value. The average TEQ_BaP_ concentration of Σ_16_PAHs in this study was higher than those in the other cities worldwide, such as Changzhi (458.92 ng g^−1^), China^33^, Beijing (49 ng g^−1^), China^19^, Delhi (154.12 ng g^−1^), India^48^, and Gwangju city (14.3 ng g^−1^), Korea^49^ Consequently, it can be stated that the PAHs concentrations in the vegetable soils of Changchun suburb might pose potential harm to the human body.

**Table 4:** TEF values for PAHs and the corresponding TEQ_BaP_ concentrations.

PAHs	TEQ_BaP_ (ng g^−1^)			
	TEF	Min	Max	Mean
Nap	0.001	0.003	0.23	0.034
Acy	0.001	0.007	0.619	0.087
Ace	0.001	0.01	0.239	0.047
Flu	0.001	0.008	0.962	0.089
Phe	0.001	0.057	1.5	0.473
Ant	0.01	0.442	7.76	2.457
Fla	0.001	0.087	1.594	0.372
Pyr	0.001	0.164	1.389	0.593
BaA	0.1	13.34	201.6	48.04
Chr	0.01	0.807	61.8	17.19
BbF	0.1	3.319	122.3	33.87
BkF	0.1	0.77	318.5	45.9
BaP	1	17.44	908.2	330.5
Daha	1	8.07	1211	222.9
InP	0.1	0.844	157.8	31.05
BghiP	0.01	2.015	23.04	9.781
Σ_16_PAHs		82.75	2320	743.4
Σ_7c_PAHs		76.07	2289	729.5

#### Health risk assessment

**Figure 2:**
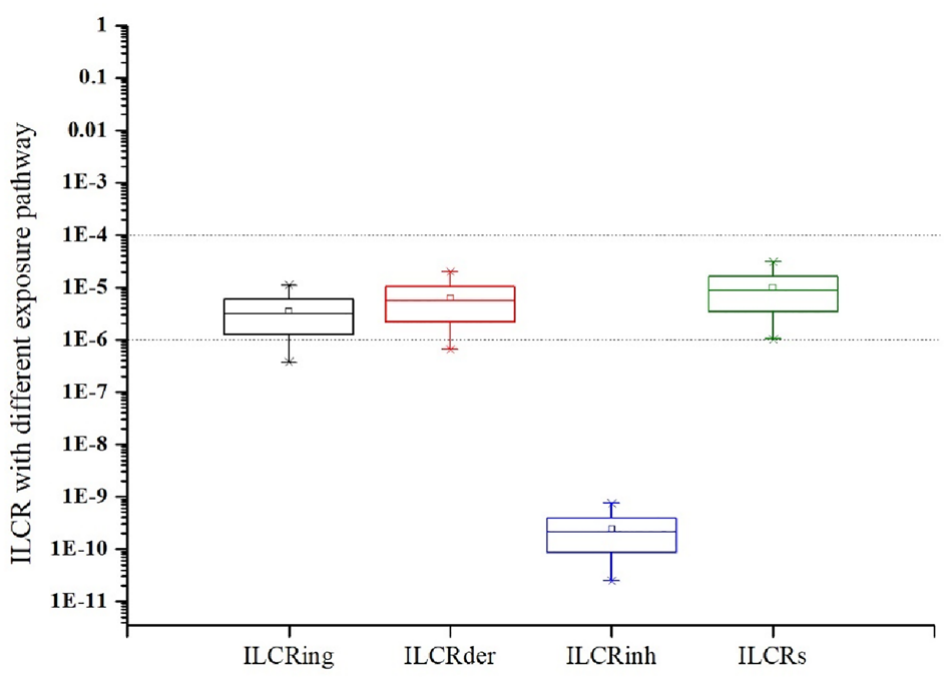
Distribution of ILCR with different exposure pathway.

The results of ILCR posed by carcinogenic PAHs in suburban vegetable soils for adults via different exposure pathways were presented in Fig. 2 and Table S3. In general, the value of ILCR less than or equal to 10^–6^ is regarded as negligible health risk. ILCR value between 10^–6^ and 10^–4^ indicates acceptable health risk or critical health level. ILCR value exceeding 10^–4^ means potential high risk, which is considered to be a serious concern and may have health problems^19^. The mean ILCRs value in all samples was 9.75 × 10^–6^, which was higher than the critical value of carcinogenic health risk. The mean values for ILCR_ing_, ILCR_der_, and ILCR_inh_ were 3.51 × 10^–6^, 6.24 × 10^–6^, and 2.37 × 10^–10^, respectively. Compared with the negligible health risk level of the inhalation route, ingestion and dermal contact exposure pathways had relatively high health risks, which were 1 × 10^4^ times higher than that of inhalation and were the main exposure pathways. The above results showed that PAHs in vegetable soils of the Changchun suburb had potential health risks for adult farmers, but the health risks were in the acceptable level. In contrast, the ILCRs values in the present study were slightly higher than that in Shanghai^50^, Beijing^19^, Gwangju, Korea^49^ and Lisbon, Portugal^51^. Therefore, PAH contamination in Changchun required more attention and further investigation should be conducted to provide more support for the assessment of PAH risks in Changchun.

## Conclusion

The Σ_16_PAHs concentrations in Changchun suburban soil were considerably higher than those in other cities around the world, and HMW PAHs were the predominant components. Source identification revealed that PAHs were mainly related to the utilization of petroleum and biomass/coal combustion. An ecological risk assessment based on the sediment quality guideline of PAHs indicated that negative ecological effects might occasionally occur in the study area. Daha and Chr had the highest proportions exceeding the ERM value, indicating that these two pollutants had the greatest negative impact on the ecosystem. According to the WHO thresholds, the TEF_Bap_ concentrations suggested that PAHs might pose a potential harm to human body. The ILCRs associated with PAHs exposure to adult farmers were acceptable and highlighted that ingestion and dermal contact were the primary exposure routes. The results obtained in this work will expand the knowledge of the effects of rapid urban development on the PAHs accumulation, and appropriate actions should be taken to prevent the occurrence of ecological risks of PAHs.

## Supplementary Information

Supplementary Tables.

## Data Availability

The datasets used and/or analyzed during the current study are available from the corresponding author on reasonable request.
