# Supplementary material for: Contamination level, sources, and health risk of polycyclic aromatic hydrocarbons in suburban vegetable field soils of Changchun, Northeast China
Source: Sci Rep. 2022 Jul 4;12:11301. doi: 10.1038/s41598-022-15285-5 (PMC9253016; doi:10.1038/s41598-022-15285-5)
Supplement: Supplementary file 1 — Supplementary Tables. [file 41598_2022_15285_MOESM1_ESM.docx]

**Contamination level, sources, and health risk of polycyclic aromatic hydrocarbons in suburban vegetable field soils of Changchun, Northeast China**

Zhengwu Cui^1,2^, Yang Wang^1^ ^*^, Liansheng Du^3^, Yong Yu ^1^

1 Northeast Institute of Geography and Agroecology, Chinese Academy of Sciences, Changchun 130102, China;

2 University of Chinese Academy of Sciences, Beijing 100049, China;

3 Shanghai Huipeng Environmental Technology Co., Ltd., Shanghai 200333, China

Corresponding author: Yang Wang. E-mail: wangyangw@iga.ac.cn

**Supplementary material**

**Table S1** Parameters of human health risk assessment

| Parameter | Symbol | Units | Adults | References |
| --- | --- | --- | --- | --- |
| Average body weight | BW | kg | 58.78 | ^1^ |
| Exposure frequency | EF | day·year^-1^ | 350 |  |
| Exposure duration | ED | year | 30 |  |
| Inhalation rate | IR_inhalation_ | m^3^·day^-1^ | 17.5 |  |
| Ingestion rate of soil | IR_ingestion_ | mg·day^-1^ | 100 |  |
| Surface area of the skin that contacts soil | SA | cm^2^·day^-1^ | 5700 |  |
| Skin adherence factor for soil | AF | mg·cm^-2^ | 0.07 |  |
| Dermal absorption factor | ABS | unitless | 0.13 |  |
| Average time | AT | day | 70×365=25550 |  |
| Particle emission factor | PEF | m^3^·kg^-1^ | 1.36×10^9^ |  |
| Carcinogenic slope factor | CSF_ingestion_ | (mg·kg^-1^·day^-1^)^-1^ | 7.3 |  |
|  | CSF_dermal_ | (mg·kg^-1^·day^-1^)^-1^ | 25 |  |
|  | CSF_inhalation_ | (mg·kg^-1^·day^-1^)^-1^ | 3.83 |  |

**Table S2** Diagnosis ratios to characterize sources of soil PAHs

| Isomeric ratios | | | Source apportionment | This study | |
| --- | --- | --- | --- | --- | --- |
|  |  |  |  | Mean | Range |
| Ant/(Phe+Ant) |  | <0.1 | Petrogenic | 0.406 | 0.079~0.804 |
|  |  | >0.1 | Pyrogenic |  |  |
|  |  |  |  |  |  |
| Flu/(Flu+Pyr) |  | <0.4 | Petroleum | 0.375 | 0.160~0.667 |
|  |  | 0.4-0.5 | Petroleum combustion |  |  |
|  |  | >0.5 | Coal, and biomass combustion |  |  |
|  |  |  |  |  |  |
| BaA/(BaA+Chr) |  | <0.2 | Petroleum | 0.356 | 0.058~0.887 |
|  |  | 0.2-0.35 | Mixed origins |  |  |
|  |  | >0.35 | Coal, and biomass combustion |  |  |
|  |  |  |  |  |  |
| InP/(InP+BghiP) |  | <0.2 | Petroleum | 0.187 | 0.009~0.560 |
|  |  | 0.2-0.5 | Mixed origins |  |  |
|  |  | >0.5 | Coal, and biomass combustion |  |  |
|  |  |  |  |  |  |
| LMW/HMW |  | >1 | Petrogenic origins | 0.185 | 0.042~0.565 |
|  |  | <1 | Pyrogenic origins |  |  |

**Table S3** Risk of cancer due to human exposure to PAHs via suburban vegetable soils of Changchun

|  | Min | Max | Mean | LCL (95%) | UCL (95%) |
| --- | --- | --- | --- | --- | --- |
| ILCR_ing_ | 3.66E-07 | 1.10E-05 | 3.51E-06 | 2.80E-06 | 4.20E-06 |
| ILCR_der_ | 6.51E-07 | 1.96E-05 | 6.24E-06 | 4.90E-06 | 7.50E-06 |
| ILCR_inh_ | 2.47E-11 | 7.44E-10 | 2.37E-10 | 1.88E-10 | 2.86E-10 |
| ILCRs | 1.02E-06 | 3.06E-05 | 9.75E-06 | 7.72E-06 | 1.18E-05 |

**Reference**

1. Chen, Y. *et al*. Accumulation characteristics and potential risk of PAHs in vegetable system grow in home garden under straw burning condition in Jilin, Northeast China. *Ecotoxicology and Environmental Safety*, **162**, 647-654. https://doi.org/10.1016/j.ecoenv.2018.06.082 (2018).
